# Pain is reduced by transcutaneous cervical vagus nerve stimulation and correlated with cardiorespiratory variability measures in the context of opioid withdrawal

**DOI:** 10.3389/fpain.2022.1031368

**Published:** 2022-11-09

**Authors:** Asim H. Gazi, Anna B. Harrison, Tamara P. Lambert, Afra Nawar, Malik Obideen, Emily G. Driggers, Viola Vaccarino, Amit J. Shah, Christopher J. Rozell, Marom Bikson, Justine W. Welsh, Omer T. Inan, J. Douglas Bremner

**Affiliations:** ^1^ School of Electrical and Computer Engineering, Georgia Institute of Technology, Atlanta, GA, United States; ^2^ Coulter Department of Biomedical Engineering, Georgia Institute of Technology, Atlanta, GA, United States; ^3^ Department of Psychiatry and Behavioral Sciences, Emory University School of Medicine, Atlanta, GA, United States; ^4^ Department of Epidemiology, Rollins School of Public Health, Emory University, Atlanta, GA, United States; ^5^ Department of Medicine, Division of Cardiology, Emory University School of Medicine, Atlanta, GA, United States; ^6^ Atlanta Veterans Affairs Health Care System, Decatur, GA, United States; ^7^ Department of Biomedical Engineering, The City College of New York, New York, NY, United States; ^8^ Department of Radiology and Imaging Sciences, Emory University School of Medicine, Atlanta, GA, United States

**Keywords:** opioid use disorder (OUD), pain, withdrawal, non-invasive, vagus nerve stimulation or VNS, heart rate variability (HRV), respiration variability, double-blind

## Abstract

Over 100,000 individuals in the United States lost their lives secondary to drug overdose in 2021, with opioid use disorder (OUD) being a leading cause. Pain is an important component of opioid withdrawal, which can complicate recovery from OUD. This study's objectives were to assess the effects of transcutaneous cervical vagus nerve stimulation (tcVNS), a technique shown to reduce sympathetic arousal in other populations, on pain during acute opioid withdrawal and to study pain's relationships with objective cardiorespiratory markers. Twenty patients with OUD underwent opioid withdrawal while participating in a two-hour protocol. The protocol involved opioid cues to induce opioid craving and neutral conditions for control purposes. Adhering to a double-blind design, patients were randomly assigned to receive active tcVNS (*n* = 9) or sham stimulation (*n* = 11) throughout the protocol. At the beginning and end of the protocol, patients' pain levels were assessed using the numerical rating scale (0–10 scale) for pain (NRS Pain). During the protocol, electrocardiogram and respiratory effort signals were measured, from which heart rate variability (HRV) and respiration pattern variability (RPV) were extracted. Pre- to post- changes (denoted with a Δ) were computed for all measures. Δ NRS Pain scores were lower (*P* = 0.045) for the active group (mean ± standard deviation: −0.8 ± 2.4) compared to the sham group (0.9 ± 1.0). A positive correlation existed between Δ NRS pain scores and Δ RPV (Spearman's *ρ* = 0.46; *P* = 0.04). Following adjustment for device group, a negative correlation existed between Δ HRV and Δ NRS Pain (Spearman's *ρ* = −0.43; *P* = 0.04). This randomized, double-blind, sham-controlled pilot study provides the first evidence of tcVNS-induced reductions in pain in patients with OUD experiencing opioid withdrawal. This study also provides the first quantitative evidence of an association between breathing irregularity and pain. The correlations between changes in pain and changes in objective physiological markers add validity to the data. Given the clinical importance of reducing pain non-pharmacologically, the findings support the need for further investigation of tcVNS and wearable cardiorespiratory sensing for pain monitoring and management in patients with OUD.

## Introduction

The opioid epidemic has been deemed a public health emergency (1). In the United States alone, over 100,000 people lost their lives to overdose in 2021—the majority due to opioids (2). Pain can present as a significant barrier in the pathway of recovery from opioid use disorder (OUD) (3, 4). Additionally, pain conditions are often a precursor to the development of OUD (5). Due to withdrawal-induced hyperalgesia, patients with OUD can experience severe pain when discontinuing opioid use (6). Although medication for OUD is meant to alleviate this burden (7), the barriers to care remain high. “Secret shopper” studies have demonstrated that fewer than 25% of patients with OUD are able to access opioid agonist medication (8). Medications like the opioid antagonist, naltrexone, require a detoxification period of 1–2 weeks prior to initiation of treatment (9, 10). This detoxification period can involve intense pain, along with other withdrawal symptoms. Novel paradigms are thus necessary for pain management when patients with OUD discontinue opioid use.

A recent study of vagus nerve stimulation (VNS) delivered percutaneously to the auricular branch of the vagus nerve observed significant reductions in opioid withdrawal symptoms within hours (11). The study also found that approximately 90% of patients using the device successfully transitioned to medication for OUD. However, the lack of any sham control and the study's open label nature did not account for potential influence of a placebo response. Moreover, no pain measurements were reported from the study, though the device was developed to target pain. A more recent sham-controlled study in healthy participants of transcutaneous cervical VNS (tcVNS) found that participants who received sham stimulation experienced a steady increase in pain scores in response to repeated noxious stimuli, while those that received active tcVNS experienced a steady decrease in pain scores (12). The study also identified brain regions that responded differently to active tcVNS compared to sham stimulation—many of which are implicated in pain (13). However, the study involved healthy participants and acute exposure to noxious stimuli, compared to chronic pain that may intensify during episodes of opioid withdrawal.

Considering the promising results of previous literature and the underlying physiology, we hypothesized that tcVNS would reduce behavioral and physiological manifestations of opioid withdrawal in patients with OUD. Our group's recent randomized, double-blind, sham-controlled pilot study evaluated this hypothesis and found statistically significant reductions in opioid withdrawal symptoms, subjective distress, and heart rate in patients receiving active tcVNS, compared to patients receiving sham stimulation (14). We also found reductions in pain for the active group compared to the sham group. However, given the broader scope of the previous article, additional pain-specific analyses were left for future work. In this study, we focus on the pain data and further elucidate the within-participant changes and between-group differences. We also address related avenues of research important to ambulatory monitoring and management of pain for patients with OUD. Specifically, we explore relationships between cardiorespiratory variability and pain and study whether these relationships possibly mediate or confound the effects of tcVNS on pain. We also assess whether tcVNS confounds the relationship between pain and cardiorespiratory variability.

## Materials and methods

### Study cohort and protocol

As part of a protocol approved by the institutional review boards of Emory University (IRB00117320) and the Georgia Institute of Technology (H20203), the psychological and physiological effects of tcVNS were studied in patients undergoing acute opioid withdrawal. Patients intending to start medication for OUD were recruited in partnership with Emory Healthcare Addiction Services and Alliance Recovery Center (Decatur, GA). To participate in the study, patients were required to be between the ages of 18 and 80 years old and meet OUD criteria according to the Structured Clinical Interview for DSM-5 (SCID). Patients who had prior experience with tcVNS, a history of carotid atherosclerosis, cervical vagotomy, schizophrenia, schizoaffective disorder, bulimia, meningitis, traumatic brain injury, neurological disorder, a loss of consciousness for greater than one minute, or any other serious medical or neurological illness were excluded. Patients were also excluded if they were implanted with a device (e.g., pacemaker), pregnant, or breastfeeding at the time. All participants provided written informed consent after receiving a complete description of the study. Data collection took place either at Alliance Recovery Center or at the Emory University School of Medicine.

**Figure 1:**
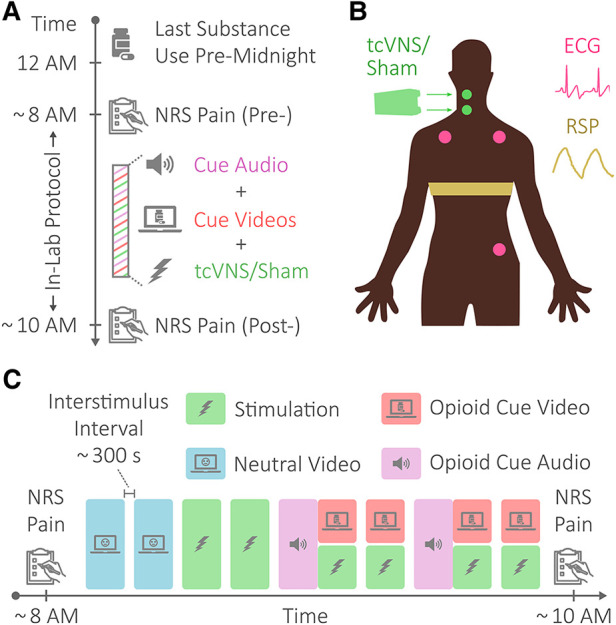
Protocol, stimulation, and physiological sensing. (**A**) Protocol overview. Patients with opioid use disorder (OUD) abstained from substance use for a minimum of eight hours prior to an in-lab protocol. The in-lab protocol consisted of opioid cue audio and videos meant to elicit opioid craving and active tcVNS or sham stimulation. (**B**) Physiological sensing and stimulation. The electrocardiogram (ECG) signal was sensed in a 3-lead configuration using Ag/AgCl electrodes placed as shown. Respiratory effort (RSP) was sensed using a resistive belt worn as shown. Transcutaneous cervical vagus nerve stimulation (tcVNS) or sham stimulation was delivered to the right side of the neck, targeting the carotid artery. (**C**) In-lab protocol. The in-lab protocol consisted of neutral videos, shown for control purposes, opioid cue audio and videos to elicit opioid craving, and tcVNS or sham stimulation. The protocol began and ended with pain measurements *via* the numerical rating scale for pain (NRS Pain).

Study participants abstained from substance use for a minimum of eight hours prior to the in-lab protocol, as illustrated in Figure 1A. The protocol lasted approximately two hours and consisted of opioid cues meant to elicit opioid craving and active tcVNS or sham stimulation (15). As outlined in Figure 1C, the in-lab protocol first involved patients viewing neutral videos meant to elicit neutral affect for control purposes (baseline). These neutral videos were played for approximately two minutes at a time and showed a mailwoman describing her job. The neutral videos were followed by administrations of active tcVNS or sham stimulation accompanied by no other stimulus. Afterwards, patients listened and watched opioid cue audio and videos, with stimulation being administered during the opioid cue videos. The opioid cue audio lasted approximately four minutes and consisted of a guided breathing exercise followed by instructions to vividly recollect recent opioid use. The opioid cue videos were played for approximately two minutes at a time and contained snippets of opioid use and imagery. Patients wore masks to protect against Coronavirus Disease 2019 (COVID-19) and remained seated in a reclining chair throughout the protocol.

### Active and sham stimulation

In this double-blind study, patients were randomly assigned to receive either active tcVNS or sham stimulation using simple randomization. The active and sham devices operated and appeared identically, differing only in the stimulation delivered. An individual not involved in recruitment, data collection, or analysis assigned pre-numbered stimulation devices to participant identification numbers. The entire dataset was then collected prior to unblinding. Patients and researchers were blinded to device group throughout screening, clinical interviews, and data collection.

For exactly two minutes at a time in accordance with the protocol shown in Figure 1C, active tcVNS or sham stimulation was administered to the right side of the neck targeting the right carotid artery, as illustrated in Figure 1B. Using a rolling switch that ranged from 0 to 5, each administration was initiated by a researcher increasing the output voltage from 0 V to the maximum level tolerable by the patient. For active tcVNS devices (gammaCore, electroCore, Basking Ridge, NJ), this corresponded to voltage amplitudes ranging from 0 to 26 V. For sham devices (gammaCore, electroCore, Basking Ridge, NJ), this corresponded to amplitudes ranging from 0 to 4 V. The active devices produced an alternating current (AC) voltage signal consisting of five periods of a 5 kHz sinusoid repeating every 40 ms, while the sham devices produced an AC biphasic square wave at the lower frequency of 0.2 Hz. Note that the output voltage of the device was not perceptible to the researcher administering stimulation. For further stimulation details, please refer to Gazi et al. (14).

### Measurement of pain

Perceived pain was measured at the beginning and end of the protocol using the Numerical Rating Scale (NRS) for pain, as shown in Figure 1. The NRS pain ranged from 0 to 10 in whole number increments. A score of 0 corresponded to no pain at all, and a score of 10 represented the worst pain ever felt. For details on other outcomes not addressed herein (e.g., measurements of withdrawal symptoms), please refer to Gazi et al. (14).

### Measurement of Heart Rate Variability (HRV) and Respiration Pattern Variability (RPV)

Electrocardiogram (ECG) and respiratory effort (RSP) signals were measured throughout the protocol at the locations shown in Figure 1B. ECG was measured using adhesive Ag/AgCl electrodes in a three-lead configuration, while RSP was transduced using a resistive belt. The Biopac RSPECR system (Biopac Systems, Goleta, CA, United States) was used for wireless data transmission, and the Biopac MP150 system was used for data acquisition at 2 kHz.

ECG signals were processed using the pre-processing, quality assessment, and feature extraction methods of prior work (16); all of the open source toolbox's default thresholds were used. For patients with detected arrhythmias (17), the toolbox's arrhythmia removal functionality was used. This produced a “clean” set of R-peak to R-peak intervals, also referred to as “normal-to-normal” (NN) intervals, that quantify the time between successive heart beats. The variability in these interval timings is used to estimate the variability in an individual's heart rate, i.e., HRV.

In this study, two well-established time domain metrics were used to quantify HRV: the standard deviation of NN intervals (SDNN) and the root mean square of successive differences (RMSSD) (18). The SDNN is a measure of total variability, as the temporal ordering of NN intervals within a time window is not considered when computing the standard deviation. RMSSD, on the other hand, focuses on the fast variations in heart rate, as it takes the differences in neighboring NN intervals and computes the root mean square of this set of differences. The PhysioNet Cardiovascular Signal Toolbox was used to estimate SDNN and RMSSD (17); and SDNN and RMSSD were calculated as in prior work using a 300 s moving window with a 299 s overlap, centered with respect to the HRV datapoint produced (16). This produced SDNN and RMSSD time series to be aggregated as desired for analysis.

RSP and ECG-derived respiration signals were processed using the pre-processing, quality assessment, and feature-level fusion methods of prior work (16); all default thresholds of the open source toolbox were used, except that the respiration quality indexing threshold was empirically tuned to 0.4 instead of 0.45 to enhance data coverage. In contrast to computations performed on a breath-by-breath basis (19), the additional averaging (described in the Analysis subsection) for RPV analysis counterbalances the added noise. The resultant inspiration time (T_i_), expiration time (T_e_), and respiration rate (RR) time series were then used to estimate RPV.

Three RPV measures were computed in this study by quantifying the coefficient of variation (CV) for the T_i_, T_e_, and RR time series. The CV is a standardized measure of dispersion computed by dividing the standard deviation by its mean and is one of the most widely used RPV metrics to date (20). As in prior work (16), the CV of T_i_, T_e_, and RR were computed using a 300 s moving window with a 299 s overlap, centered with respect to the RPV datapoint produced. This produced CV(T_i_), CV(T_e_), and CV(RR) time series to be aggregated as desired for analysis. Please refer to Supplementary Figure S1 for an illustration of the variability computation process *via* a moving window.

### Analysis

#### Comparing changes in pain scores between active and sham groups

To investigate the effects of tcVNS on opioid withdrawal-induced pain progression, the changes in the active and sham group's NRS pain scores were compared over the course of the protocol. Specifically, the initial measurement (pre-) of NRS pain was subtracted from the final measurement (post-) of NRS pain to obtain a Δ NRS pain score for each patient. These Δ NRS pain scores were compared between active and sham groups using the Mann-Whitney *U* test, after testing the data for normality using the Shapiro-Wilk test.

#### Correlating changes in pain scores with changes in HRV and RPV

To explore the relationship between changes in perceived pain and changes in cardiorespiratory variability during acute withdrawal, the Δ NRS pain scores were correlated with changes in HRV and RPV over the course of the protocol. Δ SDNN, Δ RMSSD, Δ CV(T_i_), Δ CV(T_e_), and Δ CV(RR) were each computed similarly to Δ NRS pain by subtracting a baseline value from a final value. Specifically for each metric, the average during the second neutral video was treated as the baseline value, as done for heart rate in prior work (14). The average value during the final opioid cue video and stimulation [i.e., fourth opioid cue video and sixth stimulation shown in Figure 1C] was treated as the final value. Each of these Δ variability differences were correlated with the Δ NRS pain differences across all participants using Spearman correlations, after testing the residuals of linear regression for normality using the Shapiro-Wilk test.

#### Statistical adjustments

Upon finding statistically significant results from the aforementioned analyses, additional analyses were conducted to assess for intermediate and confounding effects. Ordinal logistic regression was used instead of a Mann Whitney *U* test for the active vs. sham comparison of Δ NRS pain to adjust for Δ physiological variability. Additional adjustments for baseline physiological variability and participant characteristics were also explored. Covariate-adjusted Spearman's correlations were used to adjust for device group (i.e., active vs. sham) in the correlations between Δ physiological variability and Δ NRS pain (21). Adjustments for participant characteristics were again studied. All statistical tests performed in this study were two-tailed with a significance level of 0.05, and all analyses were performed in R.

## Results

### Patient characteristics

**Figure 2:**
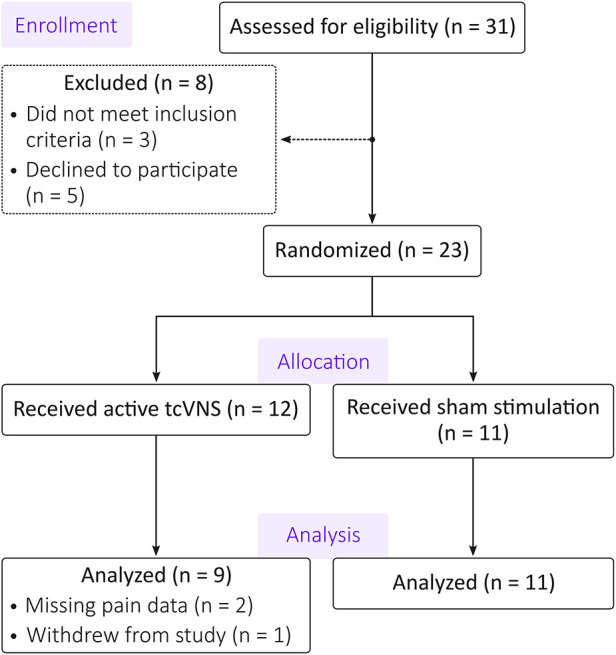
CONSORT diagram. Of the 31 patients assessed for eligibility, 8 patients were excluded or declined to participate. The remaining 23 patients were randomized to exclusively receive either active transcutaneous cervical vagus nerve stimulation (tcVNS) or sham stimulation. Two participants’ data in the active group were unusable due to equipment malfunctions. An additional participant from the active group withdrew from the study. The analysis thus included data from 9 patients in the active group and 11 patients in the sham group.

The CONSORT flow diagram for this study is shown in Figure 2. Of the 31 individuals assessed for eligibility, 23 patients were eligible and randomized. Of those 23, 12 patients received active tcVNS and 11 patients received sham stimulation. One patient in the active group withdrew from the study, and two patients' pain data were not properly stored due to equipment malfunctions. This left 9 patients with pain data available in the active group and 11 patients in the sham group for a total of *N* = 20 patients [age: 36 ± 11 years; body mass index: 28.4 ± 7.4 (mean ± standard deviation)]. Table 1 compares the active and sham groups' characteristics. Supplementary Table S1 details each patient's average stimulation amplitude. For all other characteristics, the reader is referred to Gazi et al. (14), noting that the data from patient 16 of the previous study was excluded from the present analysis due to missing NRS pain data.

**Table 1:** Active vs. sham characteristics comparison.

Parameter	Active (*n* = 9)	Sham (*n* = 11)
Age [years, mean (SD)]	38.56 (11.6)	33.27 (10.2)
Female [#, %]	1, 11%	5, 45.45%
Education [years, mean (SD)]	12.4 (0.9)	11.8 (1.4)
Studied at Alliance [#, %]	4, 44%	3, 27%
BMI [kg/m^2^, mean (SD)]	27.01 (4.77)	29.55 (9.05)
History of Smoking [#, %]	8, 89%	7, 64%
History of Alcohol Use [#, %]	5, 56%	7, 64%
History of Cardiovascular Disease [#, %]	1, 11%	1, 9%
History of Respiratory Disease [#, %]	0, 0%	1, 9%
History of Hematologic Disease [#, %]	1, 11%	0, 0%
History of Depression [#, %]	2, 22%	8, 73%
History of Posttraumatic Stress Disorder [#, %]	2, 22%	1, 9%
History of Anxiety Disorder [#, %]	1, 11%	1, 9%

SD, standard deviation.

### Reduction in Δ NRS pain for the active group compared to the sham group

**Figure 3:**
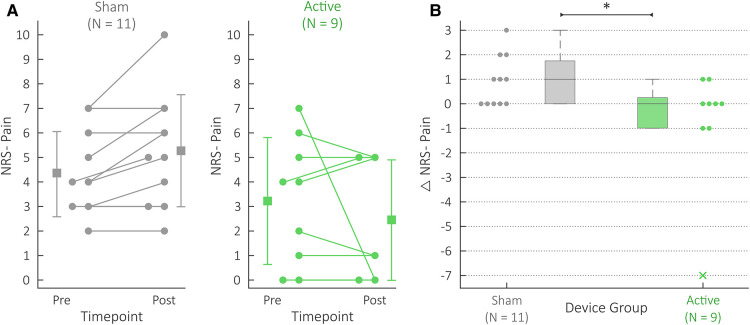
NRS pain scores for the active (green) and sham (gray) groups. (**A**) Each patient's NRS Pain datapoints. “Pre” datapoints refer to the NRS Pain scores collected at the beginning of the protocol; “Post” datapoints refer to the NRS Pain scores collected at the end of the protocol. Each patient's pair of scores is connected using a line; if two patients reported the same pain levels for both pre and post, one patient's pre and post datapoints were shifted to the left for the ease of visualization. The summary statistics depict the mean and standard deviations for the pre and post data. (**B**) Comparison of active and sham Δ NRS Pain scores. The Δ denotes differences between the post and pre NRS Pain data. Note that this Δ NRS Pain data have been presented with alternative summary statistics in previous work (14). The box and whisker plots summarize the individual Δ NRS Pain datapoints shown, where the active and sham group's data are shown on the right and left, respectively. The x marker is used for outliers, and * indicates *P* < 0.05.

The active group's Δ NRS pain scores (−0.8 ± 2.4) were significantly lower (f = 0.77; *U* = 23; *P* = 0.045) than the sham group's Δ NRS pain scores (0.9 ± 1.0). Figure 3 details this comparison. Figure 3A depicts the NRS pain scores themselves for both pre- and post- measurements, while Figure 3B summarizes the differences (i.e., Δ NRS pain scores) for both device groups. Supplementary Table S2 details the exact pre- and post- NRS pain scores for each patient.

### Positive correlation between Δ NRS pain and Δ RPV

A significant positive correlation existed between Δ NRS pain scores and Δ CV(T_i_), with a Spearman's correlation of 0.46 (*P* = 0.04). No other correlations were statistically significant unadjusted. Specifically, the Spearman's correlation between Δ NRS pain and Δ CV(T_e_) was 0.19 (*P* = 0.41), Δ CV(RR) was 0.37 (*P* = 0.11), Δ RMSSD was −0.22 (*P* = 0.35), and Δ SDNN was −0.40 (*P* = 0.08).

### Negative correlation between Δ NRS pain and Δ HRV detected following adjustment

**Figure 4:**
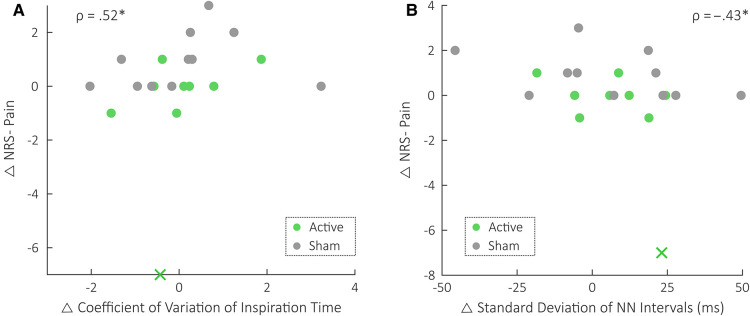
Plots of Δ NRS pain vs. Δ RPV and Δ HRV across all patients. The Spearman correlation values, *ρ*, are shown following adjustment for device group (i.e., active vs. sham). Gray is used to depict the sham group's datapoints, and green is used to depict the active group's datapoints. x indicates the outlier datapoint, and * indicates *P* < 0.05. **(A)** Plot of Δ NRS Pain vs. Δ coefficient of variation of inspiration time [CV(T_i_)]. Note that CV(T_i_) is unitless. **(B)** Plot of Δ NRS Pain vs. Δ standard deviation of Normal-to-Normal intervals (SDNN).

Table 2 details the statistics for the covariate-adjusted Spearman correlations between Δ NRS pain and Δ SDNN, adjusting for device group. As shown, a significant negative correlation existed between Δ NRS pain and Δ SDNN after adjusting for device group. This relationship is also shown in Figure 4B. The correlations between Δ NRS pain and all other variability metrics remained materially unchanged following adjustment for device group. In particular, the positive correlation between Δ NRS pain scores and Δ CV(T_i_) remained statistically significant, as detailed in Table 3. This relationship is also depicted in Figure 4A. All other device group adjustment statistics are detailed in the Supplementary Tables S3–S5. Additional adjustments for participant characteristics are detailed in Supplementary Tables S6–S10.

**Table 2:** Spearman correlations between Δ NRS pain and Δ SDNN.

Covariate Adjustments	*ρ*	95% CI	*P*
Unadjusted	−0.40	(−0.72, 0.05)	0.08
Adjusted for Device Group (i.e., Active vs. Sham)	−0.43	(−0.72, −0.02)	0.04

NRS, numeric rating scale; SDNN, standard deviation of normal to normal intervals.

**Table 3:** Spearman correlations between Δ NRS pain and Δ CV(T_i_).

Covariate Adjustments	*ρ*	95% CI	*P*
Unadjusted	0.46	(0.02, 0.75)	0.04
Adjusted for Device Group (i.e., Active vs. Sham)	0.52	(0.09, 0.79)	0.02

NRS, numeric rating scale; CV(T_i_), coefficient of variation of inspiration time.

### Reduction in Δ NRS pain remains statistically significant after adjustments

Table 4 details the results from the ordinal logistic regression comparisons between active and sham groups' Δ NRS pain scores. This analysis adjusted for Δ CV(T_i_), Δ SDNN, and both Δ CV(T_i_) and Δ SDNN simultaneously. Results did not materially change after adjusting for Δ CV(T_i_), Δ SDNN, and Δ CV(T_i_) and Δ SDNN. Likewise, results did not materially change when adjusting for participant characteristics or baseline CV(Ti) and/or baseline SDNN, as detailed in the Supplementary Table S11.

**Table 4:** Ordinal logistic regression for active vs. sham comparison of Δ NRS pain.

Covariate Adjustments	Coefficient	Standard Error	*P*
Unadjusted	−2.07	0.98	0.02
Adjusted for Δ CV (T_i_)	−2.40	1.04	0.01
Adjusted for Δ SDNN	−2.29	1.01	0.01
Adjusted for Δ CV (T_i_) and Δ SDNN	−2.41	1.05	0.01

NRS, numeric rating scale; CV(T_i_), coefficient of variation of inspiration time; SDNN, standard deviation of normal to normal intervals.

## Discussion

In this double-blind, randomized, sham-controlled pilot study, tcVNS reduced pain in patients with OUD experiencing acute opioid withdrawal. Specifically, a statistically significant decrease was observed in the difference between post-protocol and pre-protocol pain scores for patients in the active tcVNS group compared to the sham stimulation group. This study also found a statistically significant positive correlation between RPV and pain scores—the first quantitative evidence of an association between breathing irregularity and pain. HRV was also negatively correlated with pain in this data, corroborating previous literature. These correlations between changes in pain and changes in objective physiological markers add further validity to the pain measurements and their reductions *via* tcVNS. The results of this study support the need to further investigate the use of tcVNS and ambulatory cardiorespiratory sensing for pain monitoring and management in patients with OUD.

### tcVNS counteracted increases in pain during acute opioid withdrawal

In this study, tcVNS was found to counteract the potential increase in pain associated with acute opioid withdrawal. Patients in the sham group experienced increased pain or no change at all when comparing post-protocol pain measurements with those taken immediately preceding the protocol. In contrast, the majority of patients in the active group experienced decreased pain or no change at all (7 of the 9 in the active group). Although the outlier in the active group affected the mean difference observed between pre- and post- data, the statistical test ultimately used to compare the groups was rank based. Therefore, the active vs. sham difference's statistical significance was not driven by the single outlier. This tcVNS-induced reduction also cannot be attributed to the placebo effect, as the study was performed in a randomized, double-blind, sham-controlled manner. The potential for tcVNS to counteract pain during acute opioid withdrawal thus seems promising. Further investigation will be necessary to evaluate this potential in a larger sample.

This finding of a tcVNS-induced reduction in pain during acute opioid withdrawal agrees with the broader literature on tcVNS for pain management. Studies of tcVNS for pain management have generally focused on headaches and migraines (22). Numerous studies have demonstrated that tcVNS reduces the frequency and intensity of migraine attacks and cluster headaches (23–27). In fact, the United States Food and Drug Administration recently cleared tcVNS for acute and preventive treatment of pain associated with most forms of primary headache (28). Recent work has also explored tcVNS effects on noxious stimuli applied peripherally (e.g., leg) (12). This previous study found that participants who were stimulated using a sham device experienced a steady increase in pain scores each time the thermal stimulus was administered. In contrast, the group that received active tcVNS experienced a steady decrease in pain scores. This temporal interaction is interesting, as it agrees with the two-timepoint findings of the present study. The temporal interaction also suggests the use of additional timepoints of measurement in future studies of opioid withdrawal.

### Changes in respiratory variability were positively correlated with changes in pain

This analysis's positive correlation between changes in RPV and changes in perceived pain serves as the first quantitative demonstration of an association between pain and breathing irregularity. Previous studies exploring the relationship between pain and respiration have explored correspondences with parameters including respiration rate, respiration timings, inspiratory flow, and tidal volume (29–32). No analyses involving respiratory variability have been reported. Nonetheless, the patterns typically exhibited by patients in pain would explain increased RPV (33). Breath holds paired with increased inspiratory drive would explain increases in the variability of respiratory timings. Interestingly, these variations occur even under general anesthesia (34); this indicates the likely existence of an unconscious, underlying relationship between pain and RPV. The discovery of a positive correlation between RPV and pain also aligns with findings in the areas of stress and depression. Previous studies have shown that increased irregularity in respiratory rate and timings correspond to increased symptoms and neural signatures of traumatic stress in traumatized persons with and without posttraumatic stress disorder (PTSD) (16, 35). Higher RPV also predicted higher depression scores after a three-year follow-up period in patients with remitted depression (36). Others have also demonstrated similar correspondences in the context of anxiety (37, 38). This consistency across studies and domains points to a potentially generalizable relationship between increased RPV and psychologically unfavorable outcomes.

The stronger correspondence between an inspiratory RPV measure and pain scores aligns with findings reported in previous literature. Previous studies on respiration and pain have demonstrated consistently that inspiratory parameters are particularly affected by pain. Pain typically increases inspiratory flow, either by decreasing inspiration time, increasing inspiratory volume, or a combination of both (33). Inspiration is also the respiratory phase of dominant sympathetic output (39). The differing autonomic outflow during inspiration vs. expiration has been shown to be relevant to the contexts of traumatic stress, neuromodulation, and pain (40, 41). A recent study of PTSD symptoms further found that the association with variability in inspiration time was the strongest (35). Importantly, trauma is associated with chronic pain and OUD (42, 43). The specific field of RPV quantification is still nascent, however. Future work is needed to assess whether stronger positive correlations between psychologically unfavorable outcomes and inspiratory variability are generalizable.

### Changes in heart rate variability were negatively correlated with changes in pain

In agreement with previous literature on chronic pain, a negative correlation was detected between changes in HRV and changes in pain for patients undergoing opioid withdrawal. Previous studies have shown that patients suffering from spinal cord injury and neuropathic pain exhibit decreased SDNN compared to healthy controls (44). A larger cross sectional study also reported decreased HRV for those with chronic pain, as well as a negative correlation between HRV and pain for healthy participants (45). Even more compellingly, a recent meta-analysis found a significant decrease in SDNN for individuals with chronic pain compared to healthy controls (46). These negative correspondences agree with those shown in the context of stress, depression, and heart disease (47–49). Across these fields, HRV decreases are generally considered unfavorable. It is important to note, however, that this relationship may not generalize to the context of acute pain (50). In fact, the correlation between pain and HRV may even be positive in some cases (51). This may be explained by the respiratory patterns exhibited during acute pain. As aforementioned, individuals will often breathe deeply and hold their breath periodically in response to bouts of significant pain. This will increase RPV, as described above, but it could also increase HRV for a brief period or counteract a decreasing trend in HRV *via* respiratory sinus arrhythmia (52). Therefore, it is important to contextualize the negative correlation between HRV and pain of this study with findings from the pain literature involving timescales of hours, days, or longer.

### Implications for the monitoring and management of pain during opioid withdrawal

The tcVNS-induced reduction in pain and pain's correlations with cardiorespiratory variability measures are promising findings in the context of outpatient pain management during opioid withdrawal. As a non-invasive, non-pharmacological intervention that is relatively safe to use based on adverse effects data to date, the potential accessibility benefits of tcVNS are promising. This is important given the present lack of accessibility of medication for OUD (7). Non-pharmacologically reducing pain for patients undergoing opioid withdrawal could also ultimately reduce the burden of withdrawal. Pain is one of the primary risk factors for relapse in patients seeking to discontinue opioid use (3, 4). By reducing pain perceived by the patient, tcVNS could bolster a patient's ability to withstand painful periods of withdrawal on their road to recovery. Importantly, tcVNS is non-invasive and poses minimal risk (53). Outside of the clinic, patient monitoring is a challenge. Wearable physiological sensing could address this challenge by enabling continuous monitoring, requiring minimal interaction from the user, and providing objective measures. Importantly in the context of severe opioid withdrawal, a patient's mental state can be compromised, reducing the reliability of subjective measures. RPV and HRV are objective measures that can be estimated using wearable sensors [e.g., ECG and impedance pneumography (54, 55)]. A wearable system that could detect severe deterioration in a patient's pain, stress, or withdrawal state *via* measurements of RPV, HRV, and other relevant physiological parameters could inform clinicians and interventions. It is also worth noting that the effects of tcVNS on pain seem to occur *via* separate pathways from the relationship between pain and physiological measures. The statistical adjustments in all cases strengthened the relationship of interest, suggesting that the independent variables explained differing portions of the dependent variable's variance. This is favorable from the perspective of monitoring and modulation of pain, as it would imply that tcVNS would not confound the relationship between pain and physiological correlates. Thus, physiological monitoring can still be effective with or without stimulation. It is important to note, however, that this pilot study has several limitations that preclude generalization to outpatient settings over extended periods of opioid withdrawal. Future investigations will be necessary to evaluate the potential use of wearable physiological sensing and tcVNS for remote patient monitoring and management of pain.

### Limitations and future work

The study's small sample size increased the analyses' margins of error and limited the statistical power, and no power analyses were performed *a priori* to justify the sample size. The loss of two patients' data due to equipment malfunctions further reduced the size of the analytical sample. Considering the positive results of this pilot study, larger studies of tcVNS effects on pain during opioid withdrawal with adequate statistical power are warranted. Studies comparing adjunctive tcVNS use with standard of care and other interventions (e.g., alpha-2-adrenergic agonists) are also compelling. This study's discovery of a positive correlation between pain and RPV needs further validation. Additionally, the stronger correspondence between pain and inspiratory RPV measures should also be investigated. This study did not statistically test the legitimacy of the researcher and patient blinding. Future blinded experiments should administer blinding surveys to patients and researchers, as applicable, before and after the protocol. This study's results may not fully generalize to left tcVNS, as the question of right vs. left tcVNS effects remains unanswered (56). Future studies are needed to quantitatively assess whether the effects of right tcVNS and left tcVNS are similar. The measurement of pain only before and after the protocol precludes any analysis of temporal trends. Future investigations should consider the use of multiple pain measurements to better understand tcVNS effects on pain over time. As a survey with a single numeric response, NRS Pain does not provide insight into the various aspects of a patient's pain. It would be interesting in future work to investigate the components of pain [e.g., using the McGill Pain Questionnaire (57)] that change the most in response to tcVNS, and are the most associated with physiological measures. Considering this study's implications on remote patient monitoring and intervention, future work should evaluate the potential for ambulatory monitoring and management of pain using tcVNS and wearable physiological sensing.

## Conclusion

New paradigms are necessary to manage opioid withdrawal-induced pain in patients with OUD. In this double-blind, randomized, sham-controlled pilot study, tcVNS was found to counteract increases in pain perceived by patients with OUD undergoing acute opioid withdrawal. A positive correlation was also found between changes in patients' RPV and changes in their pain scores; and a negative correlation was found between changes in patients' HRV and changes in their pain scores. As a non-invasive, non-pharmacological therapy, tcVNS poses minimal risk and is amenable to self-administration. RPV and HRV can also be measured using wearable physiological sensors. This study's findings are thus promising in the context of outpatient pain management during opioid withdrawal, as they indicate the potential for ambulatory pain monitoring and management. Further investigation is necessary to evaluate the use of tcVNS and wearable cardiorespiratory sensing for remote patient monitoring and management of pain in patients with OUD.

## Data Availability

The raw data supporting the conclusions of this article will be made available by the authors, without undue reservation.
