# Supplementary material for: Pain is reduced by transcutaneous cervical vagus nerve stimulation and correlated with cardiorespiratory variability measures in the context of opioid withdrawal
Source: Front Pain Res (Lausanne). 2022 Nov 9;3:1031368. doi: 10.3389/fpain.2022.1031368 (PMC9682166; doi:10.3389/fpain.2022.1031368)
Supplement: Supplementary file 1 [file Datasheet1.docx]

Supplementary Material


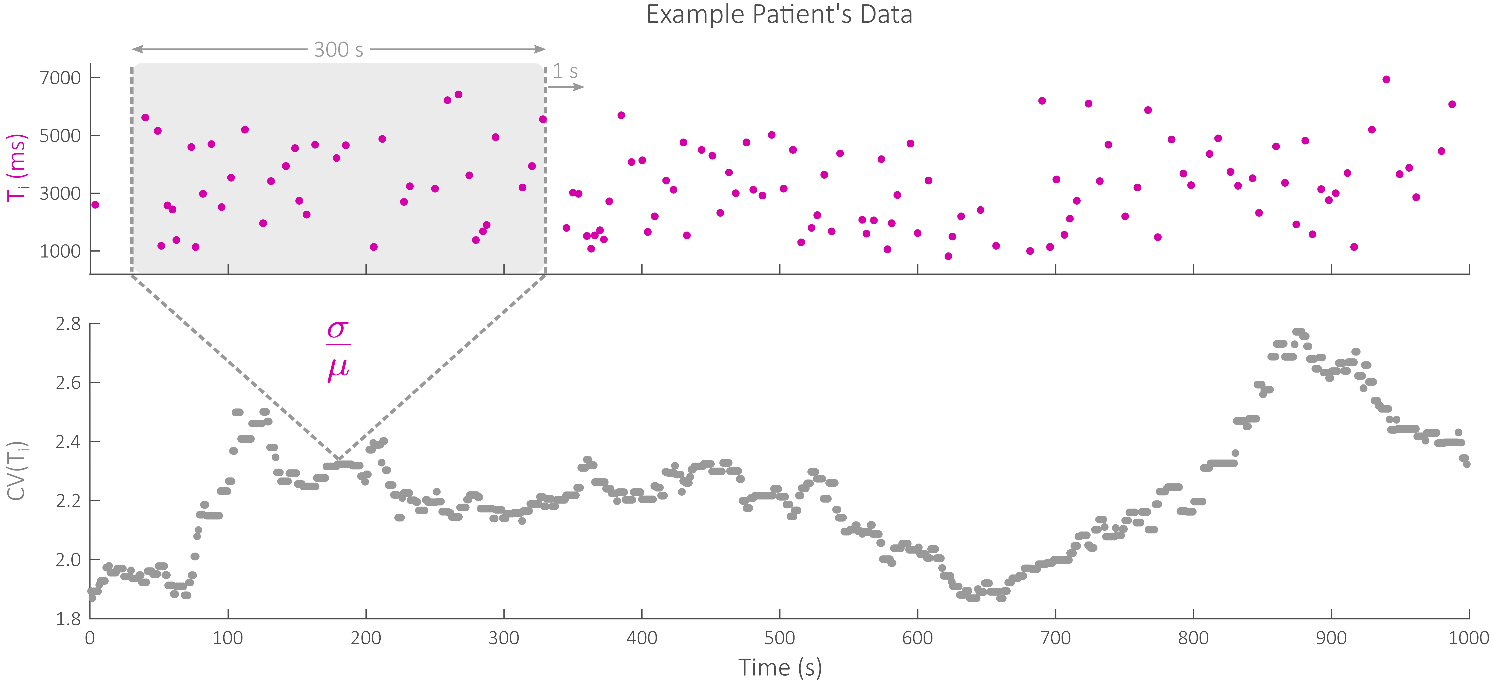


**Figure S1.** Illustration of the variability computation process via a moving window of 300 s with 299 s overlap.

**Table S1. Stimulation Amplitudes**

| **Participant Number** | **Device Group** | **Stimulation Voltage [V]** |
| --- | --- | --- |
| 1 | Sham | 4 |
| 2 | Active | 24.28 |
| 3 | Sham | 3.86 |
| 4 | Active | 7.38 |
| 5 | Sham | 3.74 |
| 6 | Sham | 4 |
| 7 | Active | 17.32 |
| 8 | Sham | 4 |
| 9 | Sham | 4 |
| 10 | Active | 16.02 |
| 11 | Sham | 4 |
| 12 | Sham | 4 |
| 13 | Sham | 4 |
| 14 | Active | 23.82 |
| 15 | Active | 20.80 |
| 16 | Sham | 3.34 |
| 17 | Active | 18.20 |
| 18 | Active | 23.40 |
| 19 | Sham | 4 |
| 20 | Active | 16.48 |

These stimulation voltages are those delivered after all intensity adjustments were made (i.e., if intensity was reduced following participant discomfort, the final intensity was recorded).

**Table S2. Pre- and Post-Protocol NRS Pain Scores**

| **Participant Number** | **Device Group** | **NRS-Pain (0-10)** | |
| --- | --- | --- | --- |
|  |  | **Pre** | **Post** |
| 1 | Sham | 4 | 5 |
| 2 | Active | 5 | 5 |
| 3 | Sham | 4 | 5 |
| 4 | Active | 0 | 0 |
| 5 | Sham | 7 | 10 |
| 6 | Sham | 3 | 3 |
| 7 | Active | 2 | 1 |
| 8 | Sham | 6 | 6 |
| 9 | Sham | 4 | 6 |
| 10 | Active | 4 | 5 |
| 11 | Sham | 3 | 3 |
| 12 | Sham | 3 | 4 |
| 13 | Sham | 2 | 2 |
| 14 | Active | 4 | 5 |
| 15 | Active | 6 | 5 |
| 16 | Sham | 5 | 7 |
| 17 | Active | 1 | 1 |
| 18 | Active | 7 | 0 |
| 19 | Sham | 7 | 7 |
| 20 | Active | 0 | 0 |

Abbreviations: NRS, numerical rating scale

**Table S3. Spearman Correlations Between Δ NRS Pain and Δ RMSSD**

| **Covariate Adjustments** | **ρ** | **95% CI** | **P** |
| --- | --- | --- | --- |
| Unadjusted | -.22 | (-.60, .25) | .35 |
| Adjusted for Device Group (i.e., Active vs. Sham) | -.24 | (-.62, .23) | .32 |

Abbreviations: NRS, numeric rating scale; RMSSD, root mean square of successive differences

**Table S4. Spearman Correlations Between Δ NRS Pain and Δ CV(T_e_)**

| **Covariate Adjustments** | **ρ** | **95% CI** | **P** |
| --- | --- | --- | --- |
| Unadjusted | .19 | (-.27, .59) | .41 |
| Adjusted for Device Group (i.e., Active vs. Sham) | .16 | (-.31, .58) | .51 |

Abbreviations: NRS, numeric rating scale; CV(T_i_), coefficient of variation of expiration time

**Table S5. Spearman Correlations Between Δ NRS Pain and Δ CV(RR)**

| **Covariate Adjustments** | **ρ** | **95% CI** | **P** |
| --- | --- | --- | --- |
| Unadjusted | .37 | (-.09, .70) | .11 |
| Adjusted for Device Group (i.e., Active vs. Sham) | .39 | (-.08, .72) | .11 |

Abbreviations: NRS, numeric rating scale; CV(RR), coefficient of variation of respiration rate

**Table S6. Spearman Correlations Between Δ NRS Pain and Δ SDNN Adjusted for Participant Characteristics**

| **Covariate Adjustments** | **ρ** | **95% CI** | **P** |
| --- | --- | --- | --- |
| Unadjusted | -.40 | (-.72, .05) | .08 |
| Adjusted for Age | -.25 | (-.61, .19) | .26 |
| Adjusted for Sex | -.12 | (-.49, .29) | .58 |
| Adjusted for Study Location | -.33 | (-.71, .20) | .21 |
| Adjusted for BMI | -.43 | (-.71, -.03) | .04 |
| Adjusted for History of Smoking | -.23 | (-.64, .29) | .39 |
| Adjusted for History of Alcohol Use | -.37 | (-.71, .12) | .13 |
| Adjusted for History of Cardiovascular Disease | -.39 | (-.65, -.07) | .02 |
| Adjusted for History of Respiratory Disease | -.37 | (-.64, -.02) | .04 |
| Adjusted for History of Hematologic Disease | -.42 | (-.67, -.08) | .02 |
| Adjusted for History of Depression | -.52 | (-.80, -.05) | .03 |
| Adjusted for History of PTSD | -.40 | (-.66, -.07) | .02 |
| Adjusted for History of Anxiety Disorder | -.28 | (-.61, .12) | .16 |

Abbreviations: NRS, numeric rating scale; SDNN, standard deviation of normal to normal intervals

**Table S7. Spearman Correlations Between Δ NRS Pain and Δ CV(T_i_) Adjusted for Participant Characteristics**

| **Covariate Adjustments** | **ρ** | **95% CI** | **P** |
| --- | --- | --- | --- |
| Unadjusted | .46 | (.02, .75) | .04 |
| Adjusted for Age | .49 | (.10, .75) | .02 |
| Adjusted for Sex | .17 | (-.35, .62) | .53 |
| Adjusted for Study Location | .42 | (-.03, .73) | .07 |
| Adjusted for BMI | .43 | (.01, .72) | .04 |
| Adjusted for History of Smoking | .28 | (-.21, .66) | .26 |
| Adjusted for History of Alcohol Use | .44 | (.03, .73) | .04 |
| Adjusted for History of Cardiovascular Disease | .45 | (.07, .71) | .02 |
| Adjusted for History of Respiratory Disease | .44 | (.06, .70) | .02 |
| Adjusted for History of Hematologic Disease | .49 | (.12, .74) | .01 |
| Adjusted for History of Depression | .55 | (.11, .81) | .02 |
| Adjusted for History of PTSD | .46 | (.10, .71) | .01 |
| Adjusted for History of Anxiety Disorder | .46 | (.08, .72) | .02 |

Abbreviations: NRS, numeric rating scale; CV(T_i_), coefficient of variation of inspiration time

**Table S8. Spearman Correlations Between Δ NRS Pain and Δ RMSSD Adjusted for Participant Characteristics**

| **Covariate Adjustments** | **ρ** | **95% CI** | **P** |
| --- | --- | --- | --- |
| Unadjusted | -.22 | (-.60, .25) | .35 |
| Adjusted for Age | -.15 | (-.56, .33) | .55 |
| Adjusted for Sex | .04 | (-.39, .46) | .85 |
| Adjusted for Study Location | -.16 | (-.65, .42) | .60 |
| Adjusted for BMI | -.27 | (-.65, .21) | .27 |
| Adjusted for History of Smoking | -.13 | (-.58, .38) | .63 |
| Adjusted for History of Alcohol Use | -.24 | (-.64, .26) | .35 |
| Adjusted for History of Cardiovascular Disease | -.21 | (-.59, .25) | .38 |
| Adjusted for History of Respiratory Disease | -.24 | (-.64, .25) | .33 |
| Adjusted for History of Hematologic Disease | -.22 | (-.62, .28) | .39 |
| Adjusted for History of Depression | -.34 | (-.70, .17) | .19 |
| Adjusted for History of PTSD | -.21 | (-.62, .28) | .40 |
| Adjusted for History of Anxiety Disorder | -.06 | (-.50, .40) | .80 |

Abbreviations: NRS, numeric rating scale; RMSSD, root mean square of successive differences

**Table S9. Spearman Correlations Between Δ NRS Pain and Δ CV(T_e_) Adjusted for Participant Characteristics**

| **Covariate Adjustments** | **ρ** | **95% CI** | **P** |
| --- | --- | --- | --- |
| Unadjusted | .19 | (-.27, .59) | .41 |
| Adjusted for Age | .14 | (-.36, .58) | .59 |
| Adjusted for Sex | .08 | (-.41, .54) | .75 |
| Adjusted for Study Location | .15 | (-.31, .56) | .52 |
| Adjusted for BMI | .20 | (-.30, .61) | .44 |
| Adjusted for History of Smoking | .22 | (-.32, .65) | .44 |
| Adjusted for History of Alcohol Use | .16 | (-.31, .56) | .52 |
| Adjusted for History of Cardiovascular Disease | .19 | (-.32, .61) | .47 |
| Adjusted for History of Respiratory Disease | .23 | (-.28, .64) | .37 |
| Adjusted for History of Hematologic Disease | .21 | (-.29, .62) | .42 |
| Adjusted for History of Depression | .13 | (-.35, .56) | .60 |
| Adjusted for History of PTSD | .19 | (-.32, .61) | .47 |
| Adjusted for History of Anxiety Disorder | .29 | (-.21, .68) | .25 |

Abbreviations: NRS, numeric rating scale; CV(T_i_), coefficient of variation of expiration time

**Table S10. Spearman Correlations Between Δ NRS Pain and Δ CV(RR) Adjusted for Participant Characteristics**

| **Covariate Adjustments** | **ρ** | **95% CI** | **P** |
| --- | --- | --- | --- |
| Unadjusted | .37 | (-.09, .70) | .11 |
| Adjusted for Age | .28 | (-.22, .66) | .27 |
| Adjusted for Sex | .18 | (-.30, .59) | .47 |
| Adjusted for Study Location | .36 | (-.10, .70) | .12 |
| Adjusted for BMI | .37 | (-.09, .70) | .11 |
| Adjusted for History of Smoking | .30 | (-.18, .67) | .22 |
| Adjusted for History of Alcohol Use | .31 | (-.19, .69) | .22 |
| Adjusted for History of Cardiovascular Disease | .34 | (-.15, .69) | .17 |
| Adjusted for History of Respiratory Disease | .38 | (-.08, .71) | .10 |
| Adjusted for History of Hematologic Disease | .37 | (-.09, .70) | .11 |
| Adjusted for History of Depression | .30 | (-.19, .67) | .23 |
| Adjusted for History of PTSD | .38 | (-.07, .70) | .09 |
| Adjusted for History of Anxiety Disorder | .42 | (-.05, .74) | .08 |

Abbreviations: NRS, numeric rating scale; CV(RR), coefficient of variation of respiration rate

**Table S11. Ordinal Logistic Regression for Active vs. Sham Comparison of Δ NRS Pain Adjusted for Participant Characteristics or Baseline CV(T_i_) and/or SDNN**

| **Covariate Adjustments** | **Coefficient** | **Standard Error** | **P** |
| --- | --- | --- | --- |
| Unadjusted | -2.07 | 0.98 | .02 |
| Adjusted for Age | -1.91 | 0.99 | .04 |
| Adjusted for Sex | -1.80 | 1.06 | .07 |
| Adjusted for Study Location | -2.01 | 0.99 | .03 |
| Adjusted for BMI | -2.09 | 0.99 | .02 |
| Adjusted for History of Smoking | -1.91 | 0.99 | .04 |
| Adjusted for History of Alcohol Use | -2.09 | 0.99 | .02 |
| Adjusted for History of Cardiovascular Disease | -2.05 | 0.98 | .02 |
| Adjusted for History of Respiratory Disease | -2.02 | 1.00 | .03 |
| Adjusted for History of Hematologic Disease | -2.83 | 1.22 | .01 |
| Adjusted for History of Depression | -1.80 | 1.06 | .07 |
| Adjusted for History of PTSD | -2.11 | 0.99 | .02 |
| Adjusted for History of Anxiety Disorder | -2.13 | 0.98 | .02 |
| Adjusted for Baseline CV(T_i_) | -2.07 | 0.98 | .02 |
| Adjusted for Baseline SDNN | -2.09 | 0.98 | .02 |
| Adjusted for Baseline CV(T_i_) and SDNN | -2.10 | 0.99 | .02 |

Abbreviations: NRS, numeric rating scale; CV(T_i_), coefficient of variation of inspiration time; SDNN, standard deviation of normal to normal intervals; PTSD, posttraumatic stress disorder
